# Supplementary material for: Tissue-specific developmental regulation and isoform usage underlie the role of doublesex in sex differentiation and mimicry in Papilio swallowtails
Source: R Soc Open Sci. 2020 Sep 30;7(9):200792. doi: 10.1098/rsos.200792 (PMC7540742; doi:10.1098/rsos.200792)

**Title:** Tissue-specific developmental regulation and isoform usage underlie the role of *doublesex* in sex differentiation and mimicry in *Papilio* swallowtails

**Authors:** Riddhi Deshmukh*, Dhanashree Lakhe and Krushnamegh Kunte*

**Affiliations:** National Centre for Biological Sciences, Tata Institute of Fundamental Research, GKVK Campus, Bellary Road, Bengaluru 560065, India.

***Corresponding author:** riddhimd@ncbs.res.in and krushnamegh@ncbs.res.in

**Supplementary information:**

Tables S1-S5

Figures S1-S2

**Table S1: Sampling details for transcriptomes sequenced across developmental stages and tissues of *Papilio polytes*.** Legend: FW: forewing, HW: hindwing, Ab: abdomen, Th: thorax.

| **Stage** |  |  | **Tissues** | | | | |  | **No. of samples** | **No. of ♂ (non-mimetic)** | **No. of ♀ (mimetic)** | **No. of libraries** |
| --- | --- | --- | --- | --- | --- | --- | --- | --- | --- | --- | --- | --- |
|  | Whole-body | Eggs | FW | HW | Ab | Th | Head | Eyes |  |  |  |  |
| Egg (2, 10, 24 hrs and 3 days after oviposition) |  | +  (5 eggs pooled) |  | |  |  |  |  | 3 at each hr/day stage |  |  | 12 |
| 1st instar larva | + (5 ind. pooled) |  |  | |  |  |  |  | 3 |  |  | 3 |
| 3rd instar larva | + |  |  | |  |  |  |  | 3 |  |  | 3 |
| 5th instar larva (late) |  |  | + | + | + | | |  | 4 | 2 | 2 | 12 |
| Pre-pupa |  |  | + | + | + | | |  | 4 | 2 | 2 | 12 |
| 3-day pupa |  |  | + | + | + | + | + |  | 4 | 2 | 2 | 20 |
| 6-day pupa |  |  | + | + | + | + | + |  | 4 | 2 | 2 | 20 |
| 9-day pupa |  |  | + | + | + | + | + |  | 4 | 2 | 2 | 20 |
| Adult female |  |  |  |  | + | + | + | + | 2 |  |  | 10 |
| Adult male |  |  |  |  | + | + | + | + | 2 |  |  | 10 |

**Table S2:** Accession details for individual raw transcriptome sequences in the NCBI SRA database. See the file “Tables2_dsx-transcriptome_SRASubmission.csv”.

**Table S3: Primers used in this study.** Added below are the isoform sequences used to design the primers.

| Targets | Forward primer (5ʹ-3ʹ) | Reverse primer (5ʹ-3ʹ) |
| --- | --- | --- |
| *dsx* (qPCR) | GCTGCAACTCACCACGCAGCGTCACA | CCGCGCTCGGAGTCGACGGAGGT |
| RPL3 | AAACTCGGCCATTTTTGTTG | GGTGGAGTCTCGATGATGGT |
| *dsx* F1 | GATAGATGAAGGAAAGCTCATCGTG | CCACCTTCGTCCGGTCATG |
| *dsx* F2 | ACAGCCCGCGGTGTCCCT | TCCATACTGGCGTGTCGAGTTC |
| *dsx* F3 | GATAGATGAAGGAAAGCTCATCGTG | ATTTCGCTCAGCATTTTCTGGCG |
| *dsx* M | CGTGGGAGATGATGCCGTTAGT | CACCCACTGAGCTTCATCTATCTT |
| *dsx* (RT-PCR) | GGCCAGCCACCGCCGGGCATA | CCGCGACGCCTCGTCGAGGTCA |

>dsxF1

ATGGTGTCCGTAGGCGCGTGGAGGCGTCGGTCTCCCGATGAATGTGACGACCGCACCGAGCCCGGGGCCTCCAGCTCAGGAGCGCCGCGTGCGCCGCCCAACTGCGCCCGTTGTCGCAACCACCGGTTGAAGGTCGAGCTGAAGGGCCACAAGCGCTACTGCAAGTACCGGTACTGCACCTGCGAGAAGTGCCGTCTCACCGCAGACCGCCAGCGCGTAATGGCTATGCAGACGGCGCTGAGGCGTGCCCAGGCGCAAGACGAGGCACGCGCGCGAGCCGCCGAGCACGGCCAGCCACCGCCGGGCATAGAGCTGGAACGGGGCGAGCCGCCTATGGTGAAGGCACTGCGAAGCCCCGTTGTACTGGCGCCTCCGGCTCCGCGCTCTCTGGCATCCTCCAGCTGTGAGTCGGTGCCCGGCTCGCCTGGTGTGTCCCCCTTCGCGCCGCCGCCGCCCTCAGTGCCGCCGCCGCCGATCATGCCGCCTCTGCTGCCGCCGCCACAGCCCGCGGTGTCCCTTGAAACCTTGGTGGAGAACTGTCACAGACTACTGGAGAAGTTCCACTACTCGTGGGAGATGATGCCGTTAGTCCTGGTCATCCTTAATTATGCTGGCAGTGACCTCGACGAGGCGTCGCGGAAGATAGATGAAGGAAAGCTAATCGTGAACGAATACGCGAGGAAGCACAATTTGAACATCTTTGATGGCCTCGAGCTGCGGAACTCGACACGCCATGACCGGACGAAGGTGGAGAAATTCGAAAAA

>dsxF2

ATGGTGTCCGTAGGCGCGTGGAGGCGTCGGTCTCCCGATGAATGTGACGACCGCACCGAGCCCGGGGCCTCCAGCTCAGGAGCGCCGCGTGCGCCGCCCAACTGCGCCCGTTGTCGCAACCACCGGTTGAAGGTCGAGCTGAAGGGCCACAAGCGCTACTGCAAGTACCGGTACTGCACCTGCGAGAAGTGCCGTCTCACCGCAGACCGCCAGCGCGTAATGGCTATGCAGACGGCGCTGAGGCGTGCCCAGGCGCAAGACGAGGCACGCGCGCGAGCCGCCGAGCACGGCCAGCCACCGCCGGGCATAGAGCTGGAACGGGGCGAGCCGCCTATGGTGAAGGCACTGCGAAGCCCCGTTGTACTGGCGCCTCCGGCTCCGCGCTCTCTGGCATCCTCCAGCTGTGAGTCGGTGCCCGGCTCGCCTGGTGTGTCCCCCTTCGCGCCGCCGCCGCCCTCAGTGCCGCCGCCGCCGATCATGCCGCCTCTGCTGCCGCCGCCACAGCCCGCGGTGTCCCTTGAAACCTTGGTGGAGAACTGTCACAGACTACTGGAGAAGTTCCACTACTCGTGGGAGATGATGCCGTTAGTCCTGGTCATCCTTAATTATGCTGGCAGTGACCTCGACGAGGCGTCGCGGAAGATAGATGAAGGAAAGCTAATCGTGAACGAATACGCGAGGAAGCACAATTTGAACATCTTTGATGGCCTCGAGCTGCGGAACTCGACACGCCAGTATGGACTTT

>dsxF3

ATGGTGTCCGTAGGCGCGTGGAGGCGTCGGTCTCCCGATGAATGTGACGACCGCACCGAGCCCGGGGCCTCCAGCTCAGGAGCGCCGCGTGCGCCGCCCAACTGCGCCCGTTGTCGCAACCACCGGTTGAAGGTCGAGCTGAAGGGCCACAAGCGCTACTGCAAGTACCGGTACTGCACCTGCGAGAAGTGCCGTCTCACCGCAGACCGCCAGCGCGTAATGGCTATGCAGACGGCGCTGAGGCGTGCCCAGGCGCAAGACGAGGCACGCGCGCGAGCCGCCGAGCACGGCCAGCCACCGCCGGGCATAGAGCTGGAACGGGGCGAGCCGCCTATGGTGAAGGCACTGCGAAGCCCCGTTGTACTGGCGCCTCCGGCTCCGCGCTCTCTGGCATCCTCCAGCTGTGAGTCGGTGCCCGGCTCGCCTGGTGTGTCCCCCTTCGCGCCGCCGCCGCCCTCAGTGCCGCCGCCGCCGATCATGCCGCCTCTGCTGCCGCCGCCACAGCCCGCGGTGTCCCTTGAAACCTTGGTGGAGAACTGTCACAGACTACTGGAGAAGTTCCACTACTCGTGGGAGATGATGCCGTTAGTCCTGGTCATCCTTAATTATGCTGGCAGTGACCTCGACGAGGCGTCGCGGAAGATAGATGAAGGAAAGCTAATCGTGAACGAATACGCGAGGAAGCACAATTTGAACATCTTTGATGGCCTCGAGCTGCGGAACTCGACACGCCAGAAAATGCTGAGCGAAATAAATAATATAAGTGGTGTAGTATCGTCTTCTATGAAGTTGTTTTGCGAAT

>dsxM

ATGGTGTCCGTAGGCGCGTGGAGGCGTCGGTCTCCCGATGAATGTGACGACCGCACCGAGCCCGGGGCCTCCAGCTCAGGAGCGCCGCGTGCGCCGCCCAACTGCGCCCGTTGTCGCAACCACCGGTTGAAGGTCGAGCTGAAGGGCCACAAGCGCTACTGCAAGTACCGGTACTGCACCTGCGAGAAGTGCCGTCTCACCGCAGACCGCCAGCGCGTAATGGCTATGCAGACGGCGCTGAGGCGTGCCCAGGCGCAAGACGAGGCACGCGCGCGAGCCGCCGAGCACGGCCAGCCACCGCCGGGCATAGAGCTGGAACGGGGCGAGCCGCCTATGGTGAAGGCACTGCGAAGCCCCGTTGTACTGGCGCCTCCGGCTCCGCGCTCTCTGGCATCCTCCAGCTGTGAGTCGGTGCCCGGCTCGCCTGGTGTGTCCCCCTTCGCGCCGCCGCCGCCCTCAGTGCCGCCGCCGCCGATCATGCCGCCTCTGCTGCCGCCGCCACAGCCCGCGGTGTCCCTTGAAACCTTGGTGGAGAACTGTCACAGACTACTGGAGAAGTTCCACTACTCGTGGGAGATGATGCCGTTAGTCCTGGTCATCCTTAATTATGCTGGCAGTGACCTCGACGAGGCGTCGCGGAAGATAGATGAAGCTCAGTGGGTGGTGCACCAGTGGCGGTTGTACGAGCGGTCGCTGTGCTCGTTGCTGGAGCTACAAGCACGCAAGGGCACGTACTGCTGTTCTTCTCAGTACGTGCTCACTCCGCAGTACGACCAGCACCTGCCGCTGCAACTCACCACGCAGCGTCACACGCCCCCGCCCGCGCACTTG

**Table S4. Details of genes co-expressed with *dsx* in mimetic (female) wings, female abdomen, non-mimetic (male) wings and male abdomen.** See file “TableS4_WGCNA_withCorrelationCoeff.csv”. Uncharacterized loci from this file were not used while constructing pie charts in Fig. 2.

**Table S5: List of genes co-expressed with *dsx* that contain *dsx* binding motif.** See the file “TableS5_WGCNATargetsWMotif.csv”.

**Table S6: Isoforms of *dsx* expressed through developmental stages and tissues of *Papilio polytes*.** Isoforms F1-F3 are female specific and M is male specific.

| **Stage** |  | **dsxF1** | **dsxF2** | **dsxF3** | **dsxM** |
| --- | --- | --- | --- | --- | --- |
| Eggs | 0h |  | + | + | ++ |
|  | 2h |  | + | + | ++ |
|  | 6h |  | + | + | ++ |
|  | 12h |  | + | + |  |
|  | 18h |  | + | + | ++ |
|  | 24h |  | + | + | ++ |
|  | 30h |  | + | + | ++ |
|  | 36h |  | + | + | ++ |
| 1st Instar | 1L |  |  |  | ++ |
| 3rd Instar | 3L |  |  |  | ++ |
| 5th Instar | FB |  | + | + | + |
|  | FFW | + | + | + | + |
|  | FHW |  | + | + | + |
|  | MB |  | + | + | ++ |
|  | MFW |  | + | + | ++ |
|  | MHW |  | + | + | ++ |
| Pre-pupae | FB |  | + | + | + |
|  | FFW |  | + | + | + |
|  | FHW |  | + | + | + |
|  | MB |  | + | + | ++ |
|  | MFW |  | + | + | ++ |
|  | MHW |  | + | + | ++ |
| 3-days post pupation | FAb |  | + | + | + |
|  | FFW | + | + | + | + |
|  | FHW | + | + | + | + |
|  | FH |  | + | + | + |
|  | FTh |  | + | + | + |
|  | MAb |  | + | + | ++ |
|  | MFW |  | + | + | ++ |
|  | MHW |  | + | + | ++ |
|  | MH |  | + | + | ++ |
|  | MTh |  | + | + | ++ |
| 6-days post pupation | FAb | + | + | + | + |
|  | FFW | + | + | + | + |
|  | FHW |  | + | + | + |
|  | FH |  | + | + | + |
|  | FTh |  | + | + | + |
|  | MAb |  | + | + | ++ |
|  | MFW |  | + | + | ++ |
|  | MHW |  | + | + | ++ |
|  | MH |  | + | + | ++ |
|  | MTh |  | + | + | ++ |
| 9-days post pupation | FAb | + | + | + |  |
|  | FFW |  | + | + |  |
|  | FHW |  | + | + |  |
|  | FH |  | + | + |  |
|  | FTh |  | + | + |  |
|  | MAb |  | + | + | ++ |
|  | MFW |  | + | + | ++ |
|  | MHW |  | + | + | ++ |
|  | MH |  | + | + | ++ |
|  | MTh |  | + | + | ++ |

**Figure S1: Differential expression between males and females 3-days after pupation.** FW (forewings), HW (hindwings), H (head), Th (thorax) and Ab (abdomen). Two blocks highlighted here contain genes that show female-specific upregulation of expression. The yellow block shows upregulation of genes in all female tissues, and genes in the purple block are upregulated in female (mimetic, M) wings compared to male (non-mimetic, NM) wings and other tissues. These contain at least one putative Dsx binding site (denoted by *), which make good candidate genes for *dsx­* targets in mimetic wings.


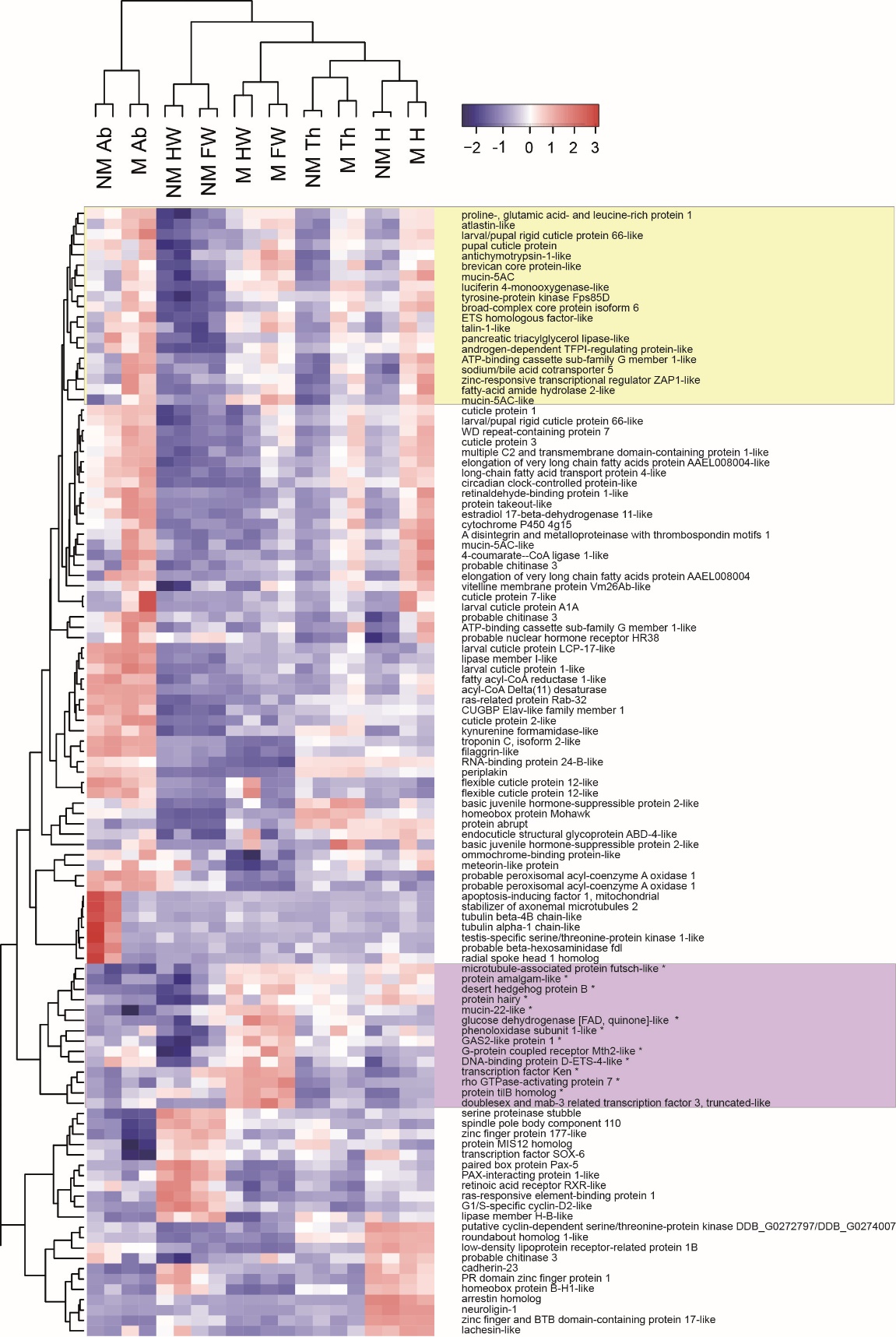


**Figure S2: Structural differences between Dsx isoforms.** The secondary structures (predicted by PSIPRED) in Dsx isoforms are shown below. The C-terminal end of each isoform differs in sequence and structure. Black bars indicate the amino acids beyond which isoforms differ.


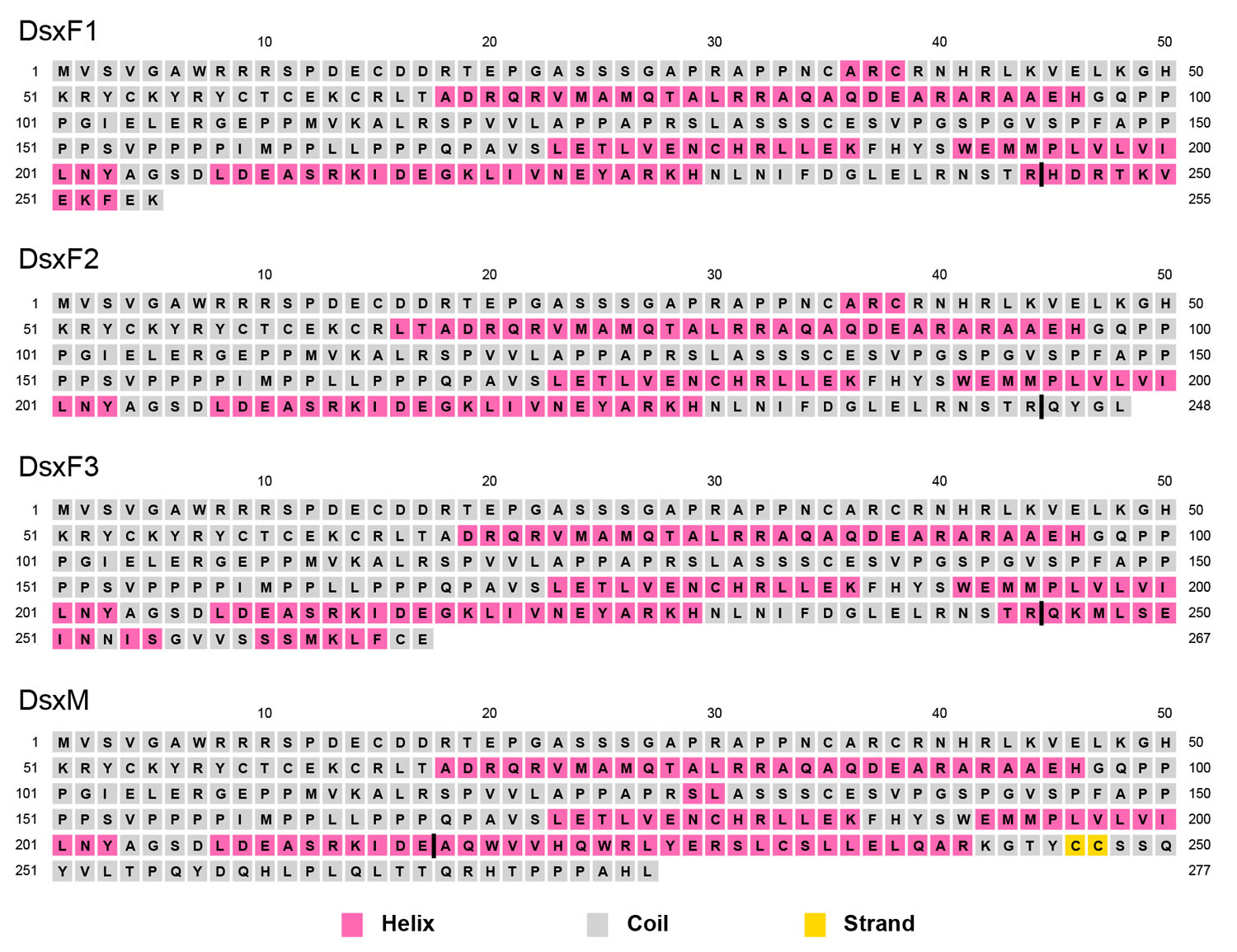

Supplement: 2020-07-15_dsxTranscriptome_SI.docx [file rsos200792supp1.docx]
